# Pandemic trends in dementia surveillance: Insights from the CCDSS, 2015 to 2023

**DOI:** 10.1002/alz70860_102915

**Published:** 2025-12-23

**Authors:** Larry C Shaver, Su‐Bin Park, Anne‐Marie Robert

**Affiliations:** ^1^ Public Health Agency of Canada, Ottawa, ON, Canada

## Abstract

**Background:**

The Canadian Chronic Disease Surveillance System (CCDSS) is a collaborative network of provincial and territorial surveillance systems in Canada. The COVID‐19 pandemic led to changes in health status, healthcare access, and health‐seeking behaviours, potentially influencing the health data used for surveillance. Existing literature highlights an initial decline in dementia case capture during the pandemic, which began to recover by late 2020. Additionally, excess mortality among people living with dementia (especially those in long‐term care settings) has been documented. This study describes national dementia surveillance data from 2015 to 2023.

**Method:**

The CCDSS links provincial and territorial health insurance registries to health administrative data in Canada.(e.g., hospitalization records, physician billings, and drug prescriptions). Using validated case definitions, the CCDSS produces annual national estimates for over 20 chronic diseases and conditions. For this study, analyses were performed to examine the average annual percentage change (AAPC) in incidence, all‐cause mortality and prevalence estimates of diagnosed dementia, including Alzheimer Disease. This was done over the five‐year pre‐pandemic period (2015 to 2020) and the three pandemic years (2020 to 2023). Estimates were age‐standardized to account for the age distribution of the population over time.

**Result:**

Comparing the AAPC in dementia estimates during the pre‐pandemic period (2015–2016 to 2019–2020) to the pandemic years (2020–2021 to 2022–2023):

Incidence: Pre‐pandemic, the incidence rate decreased by less than 1% annually, on average. In contrast, it modestly increased by 0.2% annually, on average, over the pandemic.

All‐cause mortality: Pre‐pandemic, the all‐cause mortality rate increased by less than 1% annually, on average. In contrast, it increased by 3% annually, on average, over the pandemic.

Prevalence: Pre‐pandemic, the prevalence decreased by 1% annually, on average. This decrease accelerated to 1.6% annually, on average, over the pandemic.

**Conclusion:**

The accelerated drop in the prevalence of dementia was likely driven by the increased all‐cause mortality over the pandemic period and the decrease in incidence in the first year of the pandemic. As additional data years become available, it will be possible to continue exploring the effects of the pandemic on dementia surveillance.

